# Mitochondria-DNA copy-number in osteoporosis and osteoarthritis among middle-aged women - A population-based cohort study

**DOI:** 10.1016/j.ocarto.2024.100501

**Published:** 2024-07-05

**Authors:** Christian Anker-Hansen, MirNabi Pirouzifard, Ashfaque Memon, Jan Sundquist, Kristina Sundquist, Bengt Zöller

**Affiliations:** Center for Primary Health Care Research, Lund University/Region Skåne, Malmö, Sweden

**Keywords:** Mitochondria, Epidemiology, Risk factors, Osteoporosis, Osteoarthritis

## Abstract

**Background:**

Mitochondrial DNA copy number (mtDNA-CN) is associated with aging. A relationship between mtDNA-CN and degenerative disorders, e.g. osteoarthritis (OA) and osteoporosis (OP), has been suggested. We aimed to investigate the relationship of mtDNA-CN and incident OA and OP.

**Materials and methods:**

MtDNA-CN was studied in relationship to incident OA and OP in a population-based cohort study of 6916 middle-aged women (52–63 years). Totally 2521 women with sufficient quality of mtDNA were analyzed. After exclusions, 1978 women remained in the study population. Four different endpoints obtained from the National Patient register were studied: 1) OA, 2) OP 3) OA surgery, and 4) OP fracture. In the multivariate model adjustments were made for potential OA and OP risk factors.

**Results:**

Women with low mtDNA-CN were older and had more activity at work. 125 women (6.32%) were affected by incident OP and 254 women (12.84%) had an OP fracture. Incident OA affected 451 women (22.80%) and 175 women (8.85%) had OA surgery. There were no associations between mtDNA-CN and incident risk of OA (Hazard ratio ​= ​1.00, 95% confidence interval 0.83–1.20), OA surgery (0.79, 0.58–1.07), OP (0.89, 0.62–1.27), or OP fracture (1.00, 0.78–1.29). However, incident OP was significantly associated with T-score (bone density), smoking, diabetes mellitus, and chronic obstructive bronchitis (COPD). OA was associated with body mass index and COPD.

**Conclusions:**

The present study suggests that mtDNA-CN, reflecting mitochondrial dysfunction, is not a major predictor for incident OA or OP. However, due to the limited study size minor associations cannot be excluded.

## 1 Introduction

Osteoarthritis (OA) and osteoporosis (OP) are two common age-related conditions affecting the musculoskeletal system [1]. Both conditions are degenerative diseases with an increasing incident with age, and both causing severe burden for the patients affected as well as substantial costs for the health system in general [1]. Degenerative diseases are characterized by impaired balance in the body's repair processes in relation to the natural degeneration that constantly occurs, thus resulting in cartilage degeneration in OA affected joints [2,3] and skeletal bone loss in OP skeleton [4]. It has been reported that there is an inverse relationship between OA and OP; i.e. individuals with OA have stronger bone mass and seldom have OP while patients with OP have weaker bone mass and seldom have OA changes in their joints [1]. The cause is unclear, though there exist many theories to explain this inverse relationship. OA patients have stronger body build and are more obese, which will burden the skeleton that will be stronger with higher bone mineral density [5]. The skeleton in OA patients will also be geometrical stronger with higher diameter of diaphyseal bones and stronger trabecular network, contributing to better strength [6]. It has also been shown that OA patients have different bone quality with increased content of growth factors such as IGF and TGF-Beta; factors required for bone repair [7]. Furthermore, in vitro studies of osteoblasts recruited from OA bone have different differentiation patterns and phenotypes [8].

Mitochondria are intracellular organelles involved in the synthesis of adenosine triphosphate (ATP), to provide energy for biochemical reactions within the cell [9]. The mechanism behind degenerative conditions is complex, but one theory is that mitochondria could play an important role since they can regulate cellular metabolism and cell cycles, i.e. the very survival of the cell [9]. Mitochondrial dysfunction is known to contribute to age related changes in cells [10,11] and may affect several pathways that have been implicated in cartilage degradation in OA and loss of osteoblasts in OP. Such mechanisms include oxidative stress, defective biosynthesis and growth response, increased cytokine-induced cell inflammation and matrix catabolism, increased cell apoptosis, and osteoclast activity [12,13].

Though studies have shown a relationship between the mitochondrial DNA copy number (mtDNA-CN) in peripheral blood and aging [14], only two small studies have investigated the association between mtDNA-CN and degeneration of cartilage in OA [15] and bone in OP [16], respectively. The OA study was a cross-sectional study, and the OP study was a case control study [15,16]. These studies found a relationship between OA and OP, respectively and decreased mtDNA-CN. In the present study we aimed to determine the association between mtDNA-CN and incident OA and OP, respectively, in a population-based study of middle-aged women in Lund (the Women's Health in the Lund Area - WHILA) [[17], [18], [19]].

## 2 Material and methods

### 2.1 Study population

The Women's Health in the Lund Area (WHILA) is a historical (retrospective) population-based cohort study from Lund in southern Sweden [[17], [18], [19]]. The WHILA cohort was used to investigate mtDNA-CN in relation to incident OA and OP. In the WHILA study all women who were born between 1935 and 1945 and living in the Lund area of Southern Sweden by 1995 (n ​= ​10,766), were invited to the study. The screening procedure took place from December 1995 until February 2000 [[17], [18], [19]]. Outcomes (OP and OA) and co-morbidities were obtained through linkage to the National Patient Register (NPR). The health screening program included a postal validated questionnaire concerning medical history, drug treatment, family history of diabetes and hypertension, menopausal status, smoking and alcohol habits, education, household, and working status, physical activity, quality of life as well as subjective physical and mental symptoms [[17], [18], [19]]. The screening consisted of a routine physical examination with standardized blood pressure measurements, bone densitometry and an extended laboratory examination. Out of the 10,766 women invited, 6917 (64.2%) had complete data sets, but in year 2014 one patient withdrew her consent to participation, leaving 6916 patients in the material. Blood samples for DNA analyses were available at baseline only and were collected midway through the study and therefore were available only for the last 3062 included participants [[17], [18], [19]]. Out of the 3062 blood sampled women, there were 541 samples with poor quality mtDNA as observed during droplet digital PCR analysis of reference gene [19]. Thus, only 2521 women with sufficient quality of mtDNA were analyzed, since these 541 women were excluded from analysis. Moreover, 106 women diagnosed with cancer at baseline were excluded. Another 72 were excluded due to having prevalent OA or OP at baseline. Finally, 365 women were excluded due to missing any or several of the variables included in the analysis. Thus, the final study size was 1978 women (Supplementary Fig. 1).

### 2.2 Laboratory measurement of mtDNA-CN

The mtDNA-CN was determined by a droplet digital PCR (ddPCR) based method for quantification of absolute copy number of mtDNA in whole blood [19], as described in detail [20]. The used ddPCR method for mtDNA determination has been optimized with intra- and inter-assay coefficient variances as 3.1% and 4.2% respectively [20].

### 2.3 Linking to national Swedish registers

We used several Swedish nationwide registers as part of our analyses. Statistics Sweden and the National Board of Health and Welfare maintain the registers used in the present study [[21], [22], [23], [24]].

The Swedish personal identity number is issued to all residents in Sweden and was used to connect individual-level data from different registers [24]. The personal identity numbers were replaced by Statistics Sweden with serial numbers to preserve anonymity. We used data from NPR, which includes all hospital discharge diagnoses from 1964 to 2015 and has nationwide coverage from 1987 and hospital outpatient diagnoses from 2001 to 2015 and the Swedish Cause of Death Register, which provides date and cause of death from 1961 to 2015 [[21], [22], [23], [24], [25]].

### 2.4 Adjusting variables

Individual characteristics used for adjustments were collected from the WHILA health questionnaire and included age, BMI, systolic blood pressure, T-Score, activity at work, activity at home and smoking.

Activity at work was defined from 1 to 3 were 1 was sedative work, 2 active work and 3 very active work. Activity at home was graded simply as active or not.

The other adjustable variables, i.e. comorbidities, including diabetes (ICD-10 E10–E14, ICD-9 250, ICD-8250), cardiovascular heart disease (CHD) (ICD-10 I20–I25, ICD-9410–414, and ICD-8410–414), stroke (ICD-10 I60–I69, ICD-9430–438, ICD-8430–438), and chronic obstructive pulmonary disease (COPD) (ICD-10 J40-J47, ICD-9490–496, ICD-8490–493) were collected from the NPR register [[21], [22], [23], [24]].

### 2.5 Definition of prevalent and incident OP, OA, OA surgery and OP fracture

All OP and OA outcomes are collected from the NPR [22]. The NPR has a general validity for inpatient diagnoses between 85 and 95% [22]. The diagnoses in the NPR are set by hospital specialist physicians. Baseline was defined as the date of inclusion. Follow up time was the time to event which was defined as incident of diagnose. We identified all diagnoses of OP, OA, surgical procedures for OA and OP fractures in NPR to cover every woman's medical history. Osteoarthritis diagnose could be in any joint. An osteoporosis fracture was defined as hip-, pelvic-, vertebra-, proximal humerus- and/or radius fracture.

Four different endpoints were defined; 1) incident diagnose of any diagnose of OP, 2) incident diagnose of any kind of OP diagnose, 3) incident diagnose of any kind of OA surgery and 4) incident diagnose of any type of OP fracture (hip-, pelvic-, vertebra-, proximal humerus- and radius fracture), since we wanted to find all patients with OA and OP. Patient with prevalent disease, i.e. before baseline, of the respective endpoints 1–4 were excluded.

The used ICD codes and procedure codes are explained in detail in Supplementary Tables 1 and 2. The definition of OP, OA and osteoporosis fracture are displayed in Supplementary Table 1. The definition of osteoarthritis surgery was a procedure code for OA surgery (KVÅ) are shown in Supplementary Table 2.

Incident osteoporosis, incident osteoarthrosis, incident osteoarthrosis surgery, and incident osteoporosis fracture is when diagnose date was later than the baseline date (December 1995 until February 2000) until end of follow up (2014-12-31).

### 2.6 Statistical analysis

Descriptive statistics for included variables were calculated. The difference between groups were assessed using *t*-test for continuous variables and chi-square test for categorical variables. Cox regression was used to investigate the association between mtDNA-CN and incident OP, incident OA, OA surgery and OP fractures. In an additional analysis the different types of osteoarthrosis were analyzed separately. Results are presented as Hazard Ratios (HRs) with 95% confidence intervals (CIs). The assumption of proportional hazards was determined by introducing a term with time and mtDNA-CN to test the assumption of proportional hazards. The assumption of proportionality over time was not violated. Cox regression models with mtDNA-CN both as a continuous variable and as a dichotomized variable according to mean (*118.59*) were analyzed. Three models for each outcome were calculated: model 1 crude, model 2 adjusted for age, model 3 additionally adjusted for BMI, systolic blood pressure, T-score, smoking, activity at home, activity at work, diabetes, CHD, stroke, and COPD. For each outcome, follow-up years were measured from baseline (December 1995 until February 2000) until the date of first registration for the diagnosis, death, or last follow-up date (2014-12-31) whichever came first. Analyses were performed in SAS version 9.4 (SAS Institute, USA). We calculated statical power using OpenEPi https://openepi.com/Menu/OE_Menu.htm.

## 3 Results

### 3.1 Descriptives of the study population

Among the 1978 postmenopausal women with a complete set of mtDNA-CN without prevalent OP and OA at baseline remaining after exclusions (Supplementary Fig. 1) the mean age was 56 years (range 52–63 years) (Table 1). The average BMI was 25.45 and approximately 1 out of 5 women smoked (19.1%). The average systolic blood pressure was 132, 10 (0.5%) patients had diabetes, 14 (0.7%) had coronary heart disease, 10 (0.5%) had suffered a stroke, and 32 (1.6%) had COPD at baseline. 1 out of 5 women smoked (19.1%).

**Table 1:** Descriptive characteristics of the study population (n ​= ​1978) and relation to mitochondria-DNA copy-number (mtDNA-CN).

		mtDNA-CN		
	**All** (n ​= ​1978)	***≤ 118.59*** (n ​= ​1031)	***>118.95*** (n ​= ​947)	p-value
**mtDNA Ratio ND1 EIF2C1**, mean (SD)	118.59 (27.09)	98.36 (15.00)	140.60 (18.91)	–
(min-max)	(36.70–340.00)	(36.70–118.00)	(119.00–340.00)	
**Incident Osteoporosis (OP)**	125 (6.32)	68 (6.60)	57 (6.02)	0.599^a^
**Incident Osteoarthritis (OA)**	451 (22.80)	236 (22.89)	215 (22.70)	0.921^a^
**Osteoarthritis surgery**	175 (8.85)	100 (9.70)	75 (7.92)	0.164^a^
**Osteoporosis fracture**	254 (12.84)	132 (12.80)	122 (12.88)	0.958^a^
**Age**, mean (SD)	56.67 (2.71)	56.79 (2.76)	56.54 (2.66)	0.044^b^
(min-max)	(52.00–63.00)	(52.00–63.00)	(52.00–63.00)	
**BMI**, mean (SD)	25.45 (3.97)	25.56 (4.05)	25.33 (3.89)	0.205^b^
(min-max)	(12.80–46.10)	(12.80–46.10)	(16.63–40.89)	
**SYST**, mean (SD)	131.75 (17.15) (80.00–230.00)	132.44 (17.68) (90.00–230.00)	130.99 (16.54) (80.00–195.00)	0.061^b^
(min-max)				
**TSCORE**, mean (SD) (min-max)	−0.89 (1.03)	−0.87 (1.02)	−0.91 (1.03)	0.338^b^
	(-40.20-2.70)	(-3.80-2.30)	(-4.20-2.70)	
**Activity at work**				
1	658 (33.27)	320 (31.04)	338 (35.69)	0.047^a^
2	780 (39.43)	410 (39.77)	370 (39.07)	
3	540 (27.30)	301 (29.19)	239 (25.24)	
**Activity at home**	883 (44.64)	446 (43.26)	437 (46.15)	0.197^a^
**Smoke**	377 (19.06)	235 (22.79)	142 (14.99)	<0.001^a^
**Diabetes**	10 (0.51)	6 (0.58)	4 (0.42)	0.617^a^
**CHD**	14 (0.71)	6 (0.58)	8 (0.84)	0.486^a^
**Stroke**	10 (0.51)	6 (0.58)	4 (0.42)	0.617^a^
**COPD**	32 (1.62)	23 (2.23)	9 (0.95)	0.024^a^

Abbreviations: SD ​= ​standard deviation, BMI ​= ​bodymass index, SYST ​= ​systolic bloodpressure, TSCORE ​= ​the number of SDs the bone density deviates from expected value, CHD ​= ​coronary heart disease, COPD ​= ​chronic obstructive pulmonary disorder. Activity at work: 1 is sedative, 2 active and 3 very active. Activity at home is active or not.

a χ^2^ ​test.

b Two-sided *t*-test. *P < 0.05* is consider significant.

The average T-score was (−0.9). Almost half (44.6 %) of the women were physically active at home and 66.7% had physically active or very active jobs, i.e. activity level 2 and 3.

The average mtDNA-CN in the group was 118.6. In the group with low mtDNA-CN (n ​= ​1031) the average copy number was 98.4 (range 36.7–118) and in the group with high mtDNA-CN (n ​= ​947) the average copy number was 140.6 (range 119.0–340.0). We found several significant differences between the group with low mtDNA-CN compared with the group with high mtDNA-CN. The subjects with low mtDNA-CN were older (p ​= ​0.044) and were more active at work (p ​= ​0.047), but there were no significant association with activity at home. Smoking was more common in the low mtDNA-CN group (p ​< ​00.001) and more women with low mtDNA-CN had COPD (p ​= ​0.024).

Totally 125 women (6.32%) had OP and 254 women (12.84%) had one or more OP fracture. OA was more common with 451 women (22.80%) and 175 women (8.85%) had surgery because of OA (Table 1). There was no association between low mtDNA-CN and OP, OP fracture, OA, and OA surgery, respectively.

### 3.2 mtDNA-CN and risk for incident OP

During the follow-up time of 16,013 person years 68 out of 1031 women were diagnosed with OP in the low mtDNA-CN group, corresponding to an OP incident rate at 4,27 (95% CI 3,35–5,39) per 1000 person years. In the high mtDNA-CN group 57 out of 947 were diagnosed with OP during the follow-up time of 14,962 person-years, corresponding to an OP incident rate of 3,81 (95% CI 2,94–4,94) per 1000 person years (Table 2).

**Table 2:** Mitochondria-DNA copy-number (mtDNA-CN) and risk of Osteoporosis (OP).

Variable	Person-years, No.	Cases, No./Persons at risk, No.	Incidence rate, cases/1000 person-years	Incidence rate ratio (95%CI)	HR (95% CI)		
					Model 1	Model 2	Model 3
mtDNA-CN *≤ 118.59*	16,013	68/1031	4.27 (3.35–5.39)	1 [Reference]	1 [Reference]	1 [Reference]	1 [Reference]
mtDNA-CN ​> ​*118.59*	14,962	57/947	3.81 (2.94–4.94)	0.90 (0.63–1.28)	0.89 (0.63–1.27)	0.90 (0.63–1.28)	0.89 (0.62–1.27)

Significance levels: ∗p ​< ​0.05, ∗∗p ​< ​0.01, ∗∗∗p ​< ​0.001.

Model 1 ​= ​crude model, Model 2 ​= ​adjusted for age, Model 3 ​= ​multivariable model adjusted for age, BMI, SYST, TSCORE, Smoke, Activity at home, Activity at work, Diabetes, CHD, Stroke and COPD.

Abbreviations: BMI ​= ​bodymass index, SYST ​= ​systolic bloodpressure, TSCORE ​= ​the number of SDs the bone density deviates from expected value, CHD ​= ​coronary heart disease, COPD ​= ​chronic obstructive pulmonary disorder. Activity at work: 1 is sedative, 2 active and 3 very active. Activity at home is active or not.

The univariable (model 1) Cox-regression showed no significant association between mtDNA-CN and incident risk for OP (HR ​= ​0.89, 95% 0.63–1.27) (Table 2). There were no significant associations in the adjusted model 2 (age) and model 3 (age and other risk factors) between mtDNA-CN and incident risk for OP (Table 2).

Among the adjusting variables smoking (HR ​= ​1.51, 95%CI 1.01–2.27), COPD (HR ​= ​3.25, 95% CI 1.18–8.93, diabetes (HR ​= ​6.96, 95% CI 2.18–22.21), and T-score (HR ​= ​0.44, 95% CI 0.36–0.53) were significantly associated with incident OP (Supplementary Table 3) in the full model 3. We also found significance for BMI in the univariable model 1 (HR ​= ​0.92, 95% CI 0.88–0.97) but not in the full model 3 (HR ​= ​0.98, 95% CI 0.92–1.03) (Supplementary Table 3).

### 3.3 mtDNA-CN and risk of incident OA

During the follow-up time of 14,753 person years 236 out of 1031 women were diagnosed with OA in the low mtDNA-CN group, corresponding to an OA incident rate at 16.00 (95% CI 14.08–18.17) per 1000 person-years. In the high mtDNA-CN group the follow-up time was 13,817 person-years and 215 out of 947 women were diagnosed with OA, corresponding to an OA incident rate of 15.56 (95% CI 13.61–17.79) per 1000 person years (Table 3).

**Table 3:** Mitochondria-DNA copy-number (mtDNA-CN) and risk of incident osteoarthritis (OA).

Variable	Person-years, No.	Cases, No./Persons at risk, No.	Incidence rate, cases/1000 person-years	Incidence rate ratio (95%CI)	HR (95% CI)		
					Model 1	Model 2	Model 3
mtDNA-CN *≤ 118.59*	14,753	236/1031	16.00 (14.08–18.17)	1 [Reference]	1 [Reference]	1 [Reference]	1 [Reference]
mtDNA-CN ​> ​*118.59*	13,817	215/947	15.56 (13.61–17.79)	0.97 (0.81–1.17)	0.97 (0.81–1.17)	0.97 (0.81–1.17)	1.00 (0.83–1.20)

Significance levels: ∗p ​< ​0.05, ∗∗p ​< ​0.01, ∗∗∗p ​< ​0.001.

Model 1 ​= ​crude model, Model 2 ​= ​adjusted for age, Model 3 ​= ​multivariable model adjusted for age, BMI, SYST, TSCORE, Smoke, Activity at home, Activity at work, Diabetes, CHD, Stroke and COPD.

Abbreviations: BMI ​= ​bodymass index, SYST ​= ​systolic bloodpressure, TSCORE ​= ​the number of SDs the bone density deviates from expected value, CHD ​= ​coronary heart disease, COPD ​= ​chronic obstructive pulmonary disorder. Activity at work: 1 is sedative, 2 active and 3 very active. Activity at home is active or not.

The univariable (model 1) Cox-regression showed no significant association between mtDNA-CN and incident risk for OA (HR ​= ​0.97, 95% 0.81–1.17) (Table 3). There were no significant associations in the adjusted model 2 (age) and model 3 (age and other risk factors) between mtDNA-CN and incident risk for OA (Table 3).

Among the adjusting variables BMI (HR ​= ​1.06, 95% CI 1.03–1.08) and COPD (HR ​= ​3.38, 95% CI 2.09–5.47) were significantly associated with incident OA (Supplementary Table 4) in the full model 3.

### 3.4 mtDNA-CN and risk of OA surgery

During the follow-up time of 15,828 person-years, 100 out of 1031 women had OA surgery in the low mtDNA-CN group, corresponding to an OA surgery incident rate at 6.32 (95% CI 5.19–7.69) per 1000 person-years. In the high mtDNA-CN group the follow-up time was 14,894 person-years and 75 out of 947 women had OA surgery, corresponding to an OA surgery incident rate of 5.04 (95% CI 4.02–6.31) per 1000 person-years (Table 4).

**Table 4:** Mitochondria-DNA copy-number (mtDNA-CN) and risk of incident Osteoarthritis (OA) surgery.

Variable	Person-years, No.	Cases, No./Persons at risk, No.	Incidence rate, cases/1000 person-years	Incidence rate ratio (95%CI)	HR (95% CI)		
					Model 1	Model 2	Model 3
mtDNA-CN *≤ 118.59*	15,828	100/1031	6.32 (5.19–7.69)	1 [Reference]	1 [Reference]	1 [Reference]	1 [Reference]
mtDNA-CN ​> ​*118.59*	14,894	75/947	5.04 (4.02–6.31)	0.80 (0.59–1.08)	0.79 (0.58–1.06)	0.80 (0.59–1.07)	0.79 (0.58–1.07)

Significance levels: ∗p ​< ​0.05, ∗∗p ​< ​0.01, ∗∗∗p ​< ​0.001.

Model 1 ​= ​crude model, Model 2 ​= ​adjusted for age, Model 3 ​= ​multivariable for age, BMI, SYST, TSCORE, Smoke, Activity at home, Activity at work, Diabetes, CHD, Stroke and COPD.

Abbreviations: BMI ​= ​bodymass index, SYST ​= ​systolic bloodpressure, TSCORE ​= ​the number of SDs the bone density deviates from expected value, CHD ​= ​coronary heart disease, COPD ​= ​chronic obstructive pulmonary disorder. Activity at work: 1 is sedative, 2 active and 3 very active. Activity at home is active or not.

The univariable (model 1) Cox-regression showed no significant association between mtDNA-CN and incident risk for OA surgery (HR ​= ​0.79, 95% 0.58–1.06) (Table 4). There were no significant associations in the adjusted model 2 (age) and model 3 (age and other risk factors) between mtDNA-CN and incident risk for OA surgery (Table 4).

Among the adjusting variables BMI (HR ​= ​1.10, 95% CI 1.06–1.14) was significantly associated with incident OA surgery (Supplementary Table 5) in the full model 3.

### 3.5 mtDNA-CN and risk of OP fracture

During the follow-up time of 15,526 person years, 132 out of 1031 women had OP fractures in the low mtDNA-CN group, corresponding to an OP fractures incident rate at 8.50 (95% CI 7.17–10,08) per 1000 person years. In the high mtDNA-CN group the follow-up time was 14,526 person years and 122 out of 947 women had OP fractures, corresponding to an OP fracture incident rate of 8.40 (95% CI 7.03–10.03) per 1000 person-years (Table 5).

**Table 5:** mtDNA-CN risk of osteoporosis fracture.

Variable	Person-years, No.	Cases, No./Persons at risk, No.	Incidence rate, cases/1000 person-years	Incidence rate ratio (95%CI)	HR (95% CI)		
					Model 1	Model 2	Model 3
mtDNA-CN *≤ 118.59*	15,526	132/1031	8.50 (7.17–10.08)	1 [Reference]	1 [Reference]	1 [Reference]	1 [Reference]
mtDNA-CN ​> ​*118.59*	14,526	122/947	8.40 (7.03–10.03)	0.99 (0.77–1.26)	0.98 (0.77–1.26)	1.00 (0.78–1.28)	1.00 (0.78–1.29)

Significance levels: ∗p ​< ​0.05, ∗∗p ​< ​0.01, ∗∗∗p ​< ​0.001.

Model 1 ​= ​crude model, Model 2 ​= ​adjusted for age, Model 3 ​= ​multivariable model additionally adjusted for age, BMI, SYST, TSCORE, Smoke, Activity at home, Activity at work, Diabetes, CHD, Stroke and COPD.

Abbreviations: BMI ​= ​bodymass index, SYST ​= ​systolic bloodpressure, TSCORE ​= ​the number of SDs the bone density deviates from expected value, CHD ​= ​coronary heart disease, COPD ​= ​chronic obstructive pulmonary disorder. Activity at work: 1 is sedative, 2 active and 3 very active. Activity at home is active or not.

The univariable (model 1) Cox-regression showed no significant association between mtDNA-CN and incident risk for OP fracture (HR ​= ​0.98, 95% 0.77–1.26) (Table 5). There were no significant associations in the adjusted model 2 (age) and model 3 (age and other risk factors) between mtDNA-CN and incident risk for OP fracture (Table 5).

Among the adjusting variables T-score (HR ​= ​0.72, 95%CI 0.63–0.82) was significantly associated with incident OP fracture (Supplementary Table 6) in the full model 3.

### 3.6 Additional analysis with mtDNA-CN as a continuous variable

In additional analyses mtDNA-CN was used as a continuous variable instead of dichotomized variables. However, there were no associations between mtDNA-CN and OP, OA, OA surgery or OP fracture (Supplementary Tables 7–10).

### 3.7 Sensitivity analysis

In a sensitivity analysis the excluded patients with cancer, prevalent disease (OP, OA, OA surgery and OP fractures), and missing values (Supplementary Fig. 1) were included (n ​= ​2524). However, no significant associations were observed in any models (1–3) for any endpoints (OP, OA, OA surgery, and OP fracture) (Supplements Tables 11–14).

### 3.8 Additional analysis of mtDNA-CN in different types of osteoarthritis

In an additional analysis mt-DNA-CN was compared with the different types of osteoarthrosis according to ICD-10 diagnosis (M15-M19): M15 ​= ​poly-osteoarthritis, M16 ​= ​osteoarthritis of the hip, M17 ​= ​osteoarthritis of the knee, M18 ​= ​osteoarthritis of the first carpometacarpal joint, and M19 ​= ​other and unspecified osteoarthritis (Supplementary Tables 15–19). However, the results were not significant.

### 3.9 Power calculations

Examples of power calculations are presented in Supplementary Table 20. For instance, using a two-sided 95% confidence interval we may detect a risk ratio of 0.7 for knee osteoarthritis with 82% statistical power.

## 4 Discussion

We could not find any significant associations between mtDNA-CN and incident OP, OA, OA surgery, and OP fracture. Thus, mtDNA-CN reflecting mitochondrial dysfunction is not a major biomarker for incident OP or OA. Only two small studies have previously been published regarding mtDNA-CN and OP (crossection study of 146 postmenopasual women) and OA (case control study of 204 OA patients and 169 healthy controls), respectively [15,16]. They found an association between mtDNA-CN and OP and OA, respectively. The present study argues against a major causal role for mtDNA-CN and mitochondrial dysfunction in OP and OA, respectively. Moreover, mtDNA-CN is not suggested to be a useful biomarker for incident OP or OA, respectively.

A possible explanation for that we did not find any association could be the rather low age at inclusion (age 52–63 years). We do not know if repeated measurement of mtDNA-CN at older age would result in a significant association with OA or OP. However, in the same cohort (WHILA study) mtDNA-CN has successfully predicted age related disorders such as cancer, arterial cardiovascular disease (CVD), heart failure, and type 2 diabetes but not venous thromboembolism (VTE) [19,[26], [27], [28], [29]]. Though many age-related disorders could be predicted by mtDNA-CN (cancer, arterial CVD, heart failure, and type 2 diabetes) other disorders may not be predicted, i.e. VTE, OP, and OA [19,[26], [27], [28], [29]].

In this population-based follow-up study, we found that 22.8% of the women had OA and 8.9% had surgery due to OA which correlates well with previous studies, suggesting that the studied material is representative for Sweden [30]. In a population based study from southern Sweden 26.6% had OA in 2012 [30]. We also found that 6.3% had OP and 12.8% had an osteoporotic fracture, which also correlates well with a previous study where 5.6% had OP in Sweden in 2019 [31]. It is well to note that most patients receive their diagnose of osteoporosis when they suffer their first osteoporosis related fracture, which is clearly shown in this study as twice as many patients has a fracture diagnose then a osteoporosis diagnose.

A limitation in the present study is that the women in this study were also in good health and few women had chronic conditions such as diabetes, CHD, stroke, and COPD. Moreover, only 19.1% smoked and 2/3 (66.73%) were active or very active at work, and almost half (44.64%) were active at home, suggesting that they could be considered physically fit. No validation has been performed for OP or OA in the NPR [22]. However, a strength is the use of the NPR that has 100% coverage and high validity (**85**–**95%)** for most diagnosis [22]. Another strength is that we also verified risk factors for OP such as smoking, COPD, T-score, and diabetes and for OA we found associations with BMI and COPD [[1], [2], [3],^5^]. A strength is also that we could confirm previous studies that mtDNA-CN decreased with increasing age, smoking, and COPD [[9], [10], [11], [12], [13], [14]]. Another strength is that we not only studied OA and OP but also OA surgery and OP fracture.

As previously described mtDNA-CN reflects mitochondrial health, and either a reduction or a elevation in mtDNA can indicate dysfunction [32,33]. A limitation to this study is the fact that we only had access to whole blood to analyze mtDNA-CN; this of course since it is a retrospective cohort study. It has been shown that both platelets and leukocyte count in a sample are important sources of variation if comparing mtDNA-CN among patients when mtDNA-CN is measured in DNA extracted from whole blood [32,33]. We selected two nuclear reference genes and two mtDNA genes thereby adjusting for variation in number of leucocytes. However, the number of platelets may still influence the results because they have no nucleus [32,33]. Unfortunately, we have no data for platelet count to adjust with. An association between platelets and prevalent osteoarthritis has been reported among Korean patients [34]. Thus, platelet levels might affect the results. This might result in a false negative association between mtDNA and osteoarthrosis. However, we believe our mtDNA-CN data are valid because we have shown an association between increasing age and lower mtDNA-CN and also smoking and lower mtDNA-CN (Table 1) confirming previous studies [35,36].

In conclusion, mtDNA-CN is not a major predictor for incident OP and OA. Thus, mtDNA-CN is not a useful biomarker for prediction of OA or OP, respectively. However, due to the limited study size minor associations cannot be ruled out and more research is needed to rule out a minor contribution from mtDNA-CN, reflecting mitochondrial dysfunction, in OP and OA.

## Author contributions

CAH: Collaborating with study design, statistical analysis, writing paper. AM: experimental study design and analysis of mtDNA-CN, writing and editing paper. JS: Writing and editing the paper. KS: Writing and editing the paper. MN: Study design, statistical analysis, writing, editing paper, and funding. BZ: Study design, statistical analysis, writing, editing paper, and funding.

## Funding

This work was supported by grants awarded to Dr Bengt Zöller by ALF funding from Region Skåne. The funders had no role in the study design; in the collection, analysis, and interpretation of data; in the writing of the report; and in the decision to submit the article for publication.

## Ethical approval

The regional ethical committee at Lund University approved the study (approval nos. 2011/494 and 2015/6) and written informed consent was given by all the participants in the study after full explanation of the purpose and all the protocols were conducted in accordance with the Helsinki Declaration and the Data Registry Inspection in Stockholm. Informed consent was obtained from all participants.

## Conflicts of interest

The authors declare that they have no competing interests.
