# Supplementary material for: Mitochondria-DNA copy-number in osteoporosis and osteoarthritis among middle-aged women - A population-based cohort study
Source: Osteoarthr Cartil Open. 2024 Jul 5;6(3):100501. doi: 10.1016/j.ocarto.2024.100501 (PMC11295846; doi:10.1016/j.ocarto.2024.100501)
Supplement: Multimedia component 1 [file mmc1.docx]

**Supplementary figure 1 and Supplementary tables 1-19**

**Supplementary figure 1**. Women health in Lund area (WHILA) study and Mitochondria-DNA copy-number in osteoporosis and osteoarthritis. Defining the study cohort: inclusion and exclusion criteria.

WHILA Participants **n = 6916**

Reasons for exclusion:

Missing value for mtDNA-CN

**n = 3854**

**n = 3062**

Low quality of mtDNA-CN

**n = 541**

**n = 2521**

Cancer at baseline

**n = 106**

**n = 2415**

OA or OP at baseline

**n = 72**

**n = 2343**

Internally missing values for at least one or more of included variables

**n = 365**

**Final study size n = 1978**

| **Supplementary Table 1. International classification of disease (ICD) codes used for definition of Osteoporosis, osteoarthrosis, and osteoporosis fractures in the National Patient Register (NPR). Follow-up included only ICD-10. ICD8 and ICD-9 codes were used only for exclusion of prevalent cases.** | | | |
| --- | --- | --- | --- |
| **Diagnose** | **ICD 8** | **ICD 9** | **ICD 10** |
| Osteoporosis | 7239 | 733A | M80-M81 |
| Polyosteoarthritis | 71303 | 715 | M15 |
| Osteoarthritis of hip | 71300 | 715 | M16 |
| Osteoarthritis of knee | 71301 | 715 | M17 |
| Osteoarthritis of first carpometacarpal joint | 71308 | 715 | M18 |
| Other and unspecified osteoarthritis | 71309, 71302 | 715 | M19 |
| **Osteoporosis fractures** | | | |
| Fracture of femur | 8200 | 8200 | S72 |
| Fracture of pubis | 8088 | 8088 | S325 |
| Fracture of lumbar spine and pelvis, Fracture of rib(s), sternum and thoracic spine | 8063, 8054 | 8063, 8054 | S32, S22 |
| Fracture of upper end of humerus | 8120 | 8120 | S422 |
| Fracture of lower end of radius | 8135 | 8135 | S525 |
|  | | | |

| **Supplementary Table 2. Surgical procedure codes for definition of osteoarthrosis surgery.** | | | |
| --- | --- | --- | --- |
| **Surgical procedure** | **OP6** | **KVÅ** |  |
| Joint prosthesis surgery hip | 8410-8414 | NFB09-NFB99 |  |
| Joint prosthesis surgery knee | 8423-8428 | NGB09-NGB99 |  |
| Joint prosthesis surgery hand | 8432 | NDB09-NDB99 |  |
| Joint prosthesis surgery shoulder | 8437 | NBB09-NBB99 |  |
| Joint prosthesis surgery elbow | 8432 | NCB09-NCB99 |  |
| Joint prosthesis surgery ancle and foot | 8436 | NHB09-NHB99 |  |
| OP6 = Operative procedures version 6; KVÅ = Kvalificerade vård åtgärder (Qualified healthcare procedures. | | | |

| **Supplementary Table 3**. Hazard Ratio for osteoporosis. (n=1978) | | | | |
| --- | --- | --- | --- | --- |
|  | **Ref-erence** | **HR (95% CI)** | | |
|  |  | **Model 1** | **Model 2** | **Model 3** |
| **mtDNA-CN** | *≤ 118.59* | 0.89 (0.63-1.27) | 0.90 (0.63-1.28) | 0.89 (0.62-1.27) |
| **Age** |  | 1.06 (1.00-1.13) | 1.06 (1.00-1.13) | 0.99 (0.92-1.06) |
| **BMI** |  | **0.92 (0.88-0.97)**** |  | 0.98 (0.92-1.03) |
| **SYST** |  | 1.00 (0.99-1.01) |  | 1.00 (0.99-1.01) |
| **TSCORE** |  | **0.43 (0.36-0.52)***** |  | **0.44 (0.36-0.53)***** |
| **Smoke** | No | **1.59 (1.07-2.37)*** |  | **1.51 (1.01-2.27)*** |
| **Activity at home**  Grad 2  Grad 3 | Grad 1 | 1.35 (0.90-2.04)  0.96 (0.59-1.56) |  | 1.32 (0.87-2.00)  0.94 (0.57-1.53) |
| **Activity at work** | No | 0.76 (0.53-1.09) |  | 0.78 (0.54-1.13) |
| **Diabetes** | No | **5.60 (1.78-17.62)**** |  | **6.96 (2.18-22.21)**** |
| **CHD** | No | NA |  | NA |
| **Stroke** | No | NA |  | NA |
| **COPD** | No | 2.30 (0.85-6.25) |  | **3.25 (1.18-8.93)*** |

Model 1 unadjusted. Model 2 mtDNA-CN adjusted for age. Model 3 additionally adjusted for BMI, SYST, TSCORE, Smoke, Activity at home, Activity at work, Diabetes, CHD, Stroke and COPD.

Abbreviations: SD=standard deviation, BMI=body mass index, SYST=systolic blood pressure, TSCORE= the number of SDs the bone density deviates from expected value, CHD=coronary heart disease, COPD=chronic obstructive pulmonary disorder. Activity at work: 1 is sedative, 2 active and 3 very active. Activity at home is active or not.

Significance levels: * p<0.05, ** p<0.01, *** p<0.001

| **Supplementary Table 4**. Hazard Ratio for osteoarthritis. (n=1978) | | | | |
| --- | --- | --- | --- | --- |
|  | **Ref-erence** | **HR (95% CI)** | | |
|  |  | **Model 1** | **Model 2** | **Model 3** |
| **mtDNA-CN** | *≤ 118.59* | 0.97 (0.81-1.17) | 0.97 (0.81-1.17) | 1.00 (0.83-1.20) |
| **Age** |  | 1.01 (0.97-1.04) | 1.01 (0.97-1.04) | 1.00 (0.96-1.04) |
| **BMI** |  | **1.06 (1.04-1.09)***** |  | **1.06 (1.03-1.08)***** |
| **SYST** |  | 1.00 (1.00-1.01) |  | 1.00 (0.99-1.00) |
| **TSCORE** |  | **1.12 (1.03-1.23)*** |  | 1.03 (0.94-1.14) |
| **Smoke** | No | 0.88 (0.69-1.13) |  | 0.91 (0.71-1.16) |
| **Activity at home**  Grad 2  Grad 3 | Grad 1 | 0.94 (0.76-1.17)  1.03 (0.82-1.31) |  | 0.95 (0.76-1.18)  1.02 (0.80-1.29) |
| **Activity at work** | No | 0.97 (0.81-1.17) |  | 1.06 (0.87-1.28) |
| **Diabetes** | No | **2.76 (1.14-6.66)*** |  | 1.68 (0.68-4.18) |
| **CHD** | No | 1.32 (0.50-3.54) |  | 1.28 (0.48-3.46) |
| **Stroke** | No | 2.15 (0.80-5.75) |  | 2.06 (0.76-5.59) |
| **COPD** | No | **4.31 (2.72-6.82)***** |  | **3.38 (2.09-5.47)***** |
| Model 1 unadjusted. Model 2 mtDNA-CN adjusted for age. Model 3 additionally adjusted for BMI, SYST, TSCORE, Smoke, Activity at home, Activity at work, Diabetes, CHD, Stroke and COPD.  Abbreviations: SD=standard deviation, BMI=body mass index, SYST=systolic blood pressure, TSCORE= the number of SDs the bone density deviates from expected value, CHD=coronary heart disease, COPD=chronic obstructive pulmonary disorder. Activity at work: 1 is sedative, 2 active and 3 very active. Activity at home is active or not.  Significance levels: * p<0.05, ** p<0.01, *** p<0.001 | | | | |

| **Supplementary Table 5**. Hazard Ratio for osteoarthritis surgery. (n=1978) | | | | |
| --- | --- | --- | --- | --- |
|  | **Ref-erence** | **HR (95% CI)** | | |
|  |  | **Model 1** | **Model 2** | **Model 3** |
| **mtDNA-CN** | *≤ 118.59* | 0.79 (0.58-1.06) | 0.80 (0.59-1.07) | 0.79 (0.58-1.07) |
| **Age** |  | 1.05 (1.00-1.11) | 1.05 (1.00-1.11) | 1.03 (0.98-1.09) |
| **BMI** |  | **1.11 (1.08-1.15)***** |  | **1.10 (1.06-1.14)***** |
| **SYST** |  | 1.01 (1.00-1.02) |  | 1.00 (0.99-1.01) |
| **TSCORE** |  | **1.16 (1.01-1.34)*** |  | 1.01 (0.87-1.18) |
| **Smoke** | No | 0.72 (0.47-1.10) |  | 0.70 (0.46-1.08) |
| **Activity at home**  Grad 2  Grad 3 | Grad 1 | 0.80 (0.56-1.15)  1.11 (0.77-1.59) |  | 0.80 (0.56-1.15)  1.00 (0.70-1.45) |
| **Activity at work** | No | 0.89 (0.66-1.20) |  | 1.03 (0.76-1.39) |
| **Diabetes** | No | **4.07 (1.30-12.75)*** |  | 2.39 (0.70-8.08) |
| **CHD** | No | 0.86 (0.12-6.14) |  | 0.82 (0.11-5.86) |
| **Stroke** | No | **3.81 (1.22-11.93)*** |  | 2.67 (0.79-9.04) |
| **COPD** | No | 1.60 (0.60-4.32) |  | 0.99 (0.36-2.72) |
| Model 1 unadjusted. Model 2 mtDNA-CN adjusted for age. Model 3 additionally adjusted for BMI, SYST, TSCORE, Smoke, Activity at home, Activity at work, Diabetes, CHD, Stroke and COPD.  Abbreviations: SD=standard deviation, BMI=body mass index, SYST=systolic blood pressure, TSCORE= the number of SDs the bone density deviates from expected value, CHD=coronary heart disease, COPD=chronic obstructive pulmonary disorder. Activity at work: 1 is sedative, 2 active and 3 very active. Activity at home is active or not.  Significance levels: * p<0.05, ** p<0.01, *** p<0.001 | | | | |

| **Supplementary Table 6**. Hazard Ratio for osteoporosis fracture. (n=1978) | | | | |
| --- | --- | --- | --- | --- |
|  | **Ref-erence** | **HR (95% CI)** | | |
|  |  | **Model 1** | **Model 2** | **Model 3** |
| **mtDNA-CN** | *≤ 118.59* | 0.98 (0.77-1.26) | 1.00 (0.78-1.28) | 1.00 (0.78-1.29) |
| **Age** |  | **1.06 (1.01-1.10)*** | **1.06 (1.01-1.10)*** | 1.03 (0.98-1.08) |
| **BMI** |  | 0.97 (0.94-1.00) |  | 1.00 (0.96-1.04) |
| **SYST** |  | 1.00 (0.99-1.01) |  | 1.00 (0.99-1.01) |
| **TSCORE** |  | **0.70 (0.62-0.79)***** |  | **0.72 (0.63-0.82)***** |
| **Smoke** | No | 1.26 (0.94-1.69) |  | 1.25 (0.93-1.69) |
| **Activity at home**  Grad 2  Grad 3 | Grad 1 | **1.37 (1.02-1.85)***  1.25 (0.90-1.73) |  | 1.34 (1.00-1.81)  1.20 (0.86-1.68) |
| **Activity at work** | No | 0.97 (0.76-1.24) |  | 0.98 (0.77-1.27) |
| **Diabetes** | No | 0.74 (0.10-5.29) |  | 0.70 (0.10-5.04) |
| **CHD** | No | 1.20 (0.30-4.83) |  | 1.20 (0.29-4.98) |
| **Stroke** | No | 1.57 (0.39-6.30) |  | 1.45 (0.35-6.09) |
| **COPD** | No | 1.68 (0.75-3.78) |  | 1.72 (0.76-3.88) |

Model 1 unadjusted. Model 2 mtDNA-CN adjusted for age. Model 3 additionally adjusted for BMI, SYST, TSCORE, Smoke, Activity at home, Activity at work, Diabetes, CHD, Stroke and COPD.Abbreviations: SD=standard deviation, BMI=body mass index, SYST=systolic blood pressure, TSCORE= the number of SDs the bone density deviates from expected value, CHD=coronary heart disease, COPD=chronic obstructive pulmonary disorder. Activity at work: 1 is sedative, 2 active and 3 very active. Activity at home is active or not.

Significance levels: * p<0.05, ** p<0.01, *** p<0.001

| **Supplementary Table 7** Hazard Ratio for osteoporosis. | | | | |
| --- | --- | --- | --- | --- |
|  | **Reference** | **HR (95% CI)** | | |
|  |  | **Model 1** | **Model 2** | **Model 3** |
| **mtDNA_Ratio_ND1_EIF2C1** |  | 1.00 (0.99-1.00) | 1.00 (0.99-1.00) | 1.00 (0.99-1.00) |
| Model 1 unadjusted. Model 2 mtDNA-CN adjusted for age. Model 3 additionally adjusted for BMI, SYST, TSCORE, Smoke, Activity at home, Activity at work, Diabetes, CHD, Stroke and COPD.  Abbreviations: SD=standard deviation, BMI=body mass index, SYST=systolic blood pressure, TSCORE= the number of SDs the bone density deviates from expected value, CHD=coronary heart disease, COPD=chronic obstructive pulmonary disorder. Activity at work: 1 is sedative, 2 active and 3 very active. Activity at home is active or not.  Significance levels: * p<0.05, ** p<0.01, *** p<0.001 | | | | |

| **Supplementary Table 8**. Hazard Ratio for osteoarthritis. | | | | |
| --- | --- | --- | --- | --- |
|  | **Reference** | **HR (95% CI)** | | |
|  |  | **Model 1** | **Model 2** | **Model 3** |
| **mtDNA_Ratio_ND1_EIF2C1** |  | 1.00 (1.00 -1.00) | 1.00 (1.00 -1.00) | 1.00 (1.00 -1.00) |
| Model 1 unadjusted. Model 2 mtDNA-CN adjusted for age. Model 3 additionally adjusted for BMI, SYST, TSCORE, Smoke, Activity at home, Activity at work, Diabetes, CHD, Stroke and COPD.  Abbreviations: SD=standard deviation, BMI=body mass index, SYST=systolic blood pressure, TSCORE= the number of SDs the bone density deviates from expected value, CHD=coronary heart disease, COPD=chronic obstructive pulmonary disorder. Activity at work: 1 is sedative, 2 active and 3 very active. Activity at home is active or not.  Significance levels: * p<0.05, ** p<0.01, *** p<0.001 | | | | |

| **Supplementary Table 9.** Hazard Ratio for osteoarthritis surgery. | | | | |
| --- | --- | --- | --- | --- |
|  | **Reference** | **HR (95% CI)** | | |
|  |  | **Model 1** | **Model 2** | **Model 3** |
| **mtDNA_Ratio_ND1_EIF2C1** |  | 1.00 (0.99 -1.00) | 1.00 (0.99 -1.00) | 1.00 (0.99 -1.00) |
| Model 1 unadjusted. Model 2 mtDNA-CN adjusted for age. Model 3 additionally adjusted for BMI, SYST, TSCORE, Smoke, Activity at home, Activity at work, Diabetes, CHD, Stroke and COPD.  Abbreviations: SD=standard deviation, BMI=body mass index, SYST=systolic blood pressure, TSCORE= the number of SDs the bone density deviates from expected value, CHD=coronary heart disease, COPD=chronic obstructive pulmonary disorder. Activity at work: 1 is sedative, 2 active and 3 very active. Activity at home is active or not.  Significance levels: * p<0.05, ** p<0.01, *** p<0.001 | | | | |

| **Supplementary Table 10**. Hazard Ratio for osteoporosis fracture. | | | | |
| --- | --- | --- | --- | --- |
|  | **Reference** | **HR (95% CI)** | | |
|  |  | **Model 1** | **Model 2** | **Model 3** |
| **mtDNA_Ratio_ND1_EIF2C1** |  | 1.00 (0.99 -1.00) | 1.00 (0.99 -1.00) | 1.00 (0.99 -1.00) |
| Model 1 unadjusted. Model 2 mtDNA-CN adjusted for age. Model 3 additionally adjusted for BMI, SYST, TSCORE, Smoke, Activity at home, Activity at work, Diabetes, CHD, Stroke and COPD.  Abbreviations: SD=standard deviation, BMI=body mass index, SYST=systolic blood pressure, TSCORE= the number of SDs the bone density deviates from expected value, CHD=coronary heart disease, COPD=chronic obstructive pulmonary disorder. Activity at work: 1 is sedative, 2 active and 3 very active. Activity at home is active or not.  Significance levels: * p<0.05, ** p<0.01, *** p<0.001 | | | | |

| **Supplementary Table 11**. Hazard Ratio for Osteoporosis. (n= 2524) | | | | |
| --- | --- | --- | --- | --- |
|  |  | **HR (95% CI)** | | |
|  |  | **Model 1** | **Model 2** | **Model 3** |
| **mtDNA_Ratio_ND1_EIF2C1** |  | 1.00 (0.99-1.00) | 1.00 (0.99-1.00) | 1.00 (0.99-1.00) |
| Model 1 unadjusted. Model 2 mtDNA-CN adjusted for age. Model 3 additionally adjusted for BMI, SYST, TSCORE, Smoke, Activity at home, Activity at work, Diabetes, CHD, Stroke and COPD.  Abbreviations: SD=standard deviation, BMI=body mass index, SYST=systolic blood pressure, TSCORE= the number of SDs the bone density deviates from expected value, CHD=coronary heart disease, COPD=chronic obstructive pulmonary disorder. Activity at work: 1 is sedative, 2 active and 3 very active. Activity at home is active or not.  Significance levels: * p<0.05, ** p<0.01, *** p<0.001 | | | | |

| **Supplementary Table 12**. Hazard Ratio for Osteoarthritis. (n= 2524) | | | | |
| --- | --- | --- | --- | --- |
|  |  | **HR (95% CI)** | | |
|  |  | **Model 1** | **Model 2** | **Model 3** |
| **mtDNA_Ratio_ND1_EIF2C1** |  | 1.00 (1.00 -1.00) | 1.00 (1.00 -1.00) | 1.00 (1.00 -1.00) |
| Model 1 unadjusted. Model 2 mtDNA-CN adjusted for age. Model 3 additionally adjusted for BMI, SYST, TSCORE, Smoke, Activity at home, Activity at work, Diabetes, CHD, Stroke and COPD.  Abbreviations: SD=standard deviation, BMI=body mass index, SYST=systolic blood pressure, TSCORE= the number of SDs the bone density deviates from expected value, CHD=coronary heart disease, COPD=chronic obstructive pulmonary disorder. Activity at work: 1 is sedative, 2 active and 3 very active. Activity at home is active or not.  Significance levels: * p<0.05, ** p<0.01, *** p<0.001 | | | | |

| **Supplementary Table 13**. Hazard Ratio for Osteoarthritis surgery. (n= 2524) | | | | |
| --- | --- | --- | --- | --- |
|  |  | **HR (95% CI)** | | |
|  |  | **Model 1** | **Model 2** | **Model 3** |
| **mtDNA_Ratio_ND1_EIF2C1** |  | 1.00 (0.99 -1.00) | 1.00 (1.00 -1.00) | 1.00 (1.00 -1.00) |
| Model 1 unadjusted. Model 2 mtDNA-CN adjusted for age. Model 3 additionally adjusted for BMI, SYST, TSCORE, Smoke, Activity at home, Activity at work, Diabetes, CHD, Stroke and COPD.  Abbreviations: SD=standard deviation, BMI=body mass index, SYST=systolic blood pressure, TSCORE= the number of SDs the bone density deviates from expected value, CHD=coronary heart disease, COPD=chronic obstructive pulmonary disorder. Activity at work: 1 is sedative, 2 active and 3 very active. Activity at home is active or not.  Significance levels: * p<0.05, ** p<0.01, *** p<0.001 | | | | |

| **Supplementary Table 14**. Hazard Ratio for Osteoporosis fracture. (n= 2524) | | | | |
| --- | --- | --- | --- | --- |
|  | **Reference** | **HR (95% CI)** | | |
|  |  | **Model 1** | **Model 2** | **Model 3** |
| **mtDNA_Ratio_ND1_EIF2C1** |  | 1.00 (1.00 -1.00) | 1.00 (1.00 -1.00) | 1.00 (1.00 -1.00) |
| Model 1 unadjusted. Model 2 mtDNA-CN adjusted for age. Model 3 additionally adjusted for BMI, SYST, TSCORE, Smoke, Activity at home, Activity at work, Diabetes, CHD, Stroke and COPD.  Abbreviations: SD=standard deviation, BMI=body mass index, SYST=systolic blood pressure, TSCORE= the number of SDs the bone density deviates from expected value, CHD=coronary heart disease, COPD=chronic obstructive pulmonary disorder. Activity at work: 1 is sedative, 2 active and 3 very active. Activity at home is active or not.  Significance levels: * p<0.05, ** p<0.01, *** p<0.001 | | | | |

| **Supplementary table 15. mtDNA-CN** **risk of M15.** | | | | | | | |
| --- | --- | --- | --- | --- | --- | --- | --- |
| Variable | Person-years,  No. | Cases, No./Persons at risk, No. | Incidence rate, cases/1000 person-years | Incidence rate ratio (95%CI) | HR(95% CI) | | |
|  |  |  |  |  | **Model 1** | **Model 2** | **Model 3** |
| mtDNA-CN *≤ 118.59* | 16075 | 44/1031 | 2.74  (2.04-3.68) | 1 [Reference] | 1 [Reference] | 1 [Reference] | 1 [Reference] |
| mtDNA-CN > *118.59* | 15083 | 30/947 | 1.99  (1.39-2.84) | 0.73  (0.46-1.16) | 0.73  (0.46-1.16) | 0.73  (0.46-1.17) | 0.73  (0.46-1.17) |
| Model 1 crude model. Model 2 adjusted for age. Model 3 additionally adjusted for BMI, SYST, TSCORE, Smoke, Activity at home, Activity at work, Diabetes, CHD, Stroke and COPD. | | | | | | | |

Abbreviations: SD=standard deviation, BMI=body mass index, SYST=systolic blood pressure, TSCORE= the number of SDs the bone density deviates from expected value, CHD=coronary heart disease, COPD=chronic obstructive pulmonary disorder. Activity at work: 1 is sedative, 2 active and 3 very active. Activity at home is active or not.

Significance levels: * p<0.05, ** p<0.01, *** p<0.001

| **Supplementary table 16**. **mtDNA-CN** **risk of M16.** | | | | | | | |
| --- | --- | --- | --- | --- | --- | --- | --- |
| Variable | Person-years,  No. | Cases, No./Persons at risk, No. | Incidence rate, cases/1000 person-years | Incidence rate ratio (95%CI) | HR(95% CI) | | |
|  |  |  |  |  | **Model 1** | **Model 2** | **Model 3** |
| mtDNA-CN *≤ 118.59* | 16030 | 68/1031 | 4.24  (3.34-5.38) | 1 [Reference] | 1 [Reference] | 1 [Reference] | 1 [Reference] |
| mtDNA-CN > *118.59* | 15002 | 51/947 | 3.40  (2.58-4.47) | 0.80  (0.56-1.15) | 0.79  (0.55-1.14) | 0.80  (0.55-1.14) | 0.77  (0.53-1.11) |
| Model 1 crude model. Model 2 adjusted for age. Model 3 additionally adjusted for BMI, SYST, TSCORE, Smoke, Activity at home, Activity at work, Diabetes, CHD, Stroke and COPD. | | | | | | | |

Abbreviations: SD=standard deviation, BMI=body mass index, SYST=systolic blood pressure, TSCORE= the number of SDs the bone density deviates from expected value, CHD=coronary heart disease, COPD=chronic obstructive pulmonary disorder. Activity at work: 1 is sedative, 2 active and 3 very active. Activity at home is active or not.

Significance levels: * p<0.05, ** p<0.01, *** p<0.001

| **Supplementary table 17**. **mtDNA-CN** **risk of M17.** | | | | | | | |
| --- | --- | --- | --- | --- | --- | --- | --- |
| Variable | Person-years,  No. | Cases, No./Persons at risk, No. | Incidence rate, cases/1000 person-years | Incidence rate ratio (95%CI) | HR(95% CI) | | |
|  |  |  |  |  | **Model 1** | **Model 2** | **Model 3** |
| mtDNA-CN *≤ 118.59* | 15795 | 99/1031 | 6.27  (5.15-7.63) | 1 [Reference] | 1 [Reference] | 1 [Reference] | 1 [Reference] |
| mtDNA-CN > *118.59* | 14699 | 100/947 | 6.80  (5.59-8.28) | 1.09  (0.82-1.43) | 1.08  (0.82-1.43) | 0.92  (0.69-1.21) | 1.13  (0.85-1.50) |
| Model 1 crude model. Model 2 adjusted for age. Model 3 additionally adjusted for BMI, SYST, TSCORE, Smoke, Activity at home, Activity at work, Diabetes, CHD, Stroke and COPD. | | | | | | | |

Abbreviations: SD=standard deviation, BMI=body mass index, SYST=systolic blood pressure, TSCORE= the number of SDs the bone density deviates from expected value, CHD=coronary heart disease, COPD=chronic obstructive pulmonary disorder. Activity at work: 1 is sedative, 2 active and 3 very active. Activity at home is active or not.

Significance levels: * p<0.05, ** p<0.01, *** p<0.001

| **Supplementary table 18**. **mtDNA-CN** **risk of M18.** | | | | | | | |
| --- | --- | --- | --- | --- | --- | --- | --- |
| Variable | Person-years,  No. | Cases, No./Persons at risk, No. | Incidence rate, cases/1000 person-years | Incidence rate ratio (95%CI) | HR(95% CI) | | |
|  |  |  |  |  | **Model 1** | **Model 2** | **Model 3** |
| mtDNA-CN *≤ 118.59* | 16048 | 44/1031 | 2.74  (2.04-3.68) | 1 [Reference] | 1 [Reference] | 1 [Reference] | 1 [Reference] |
| mtDNA-CN > *118.59* | 15050 | 40/947 | 2.66  (1.95-3.62) | 0.97  (0.62-1.49) | 0.97  (0.63-1.49) | 0.96  (0.63-1.48) | 0.96  (0.62-1.47) |
| Model 1 crude model. Model 2 adjusted for age. Model 3 additionally adjusted for BMI, SYST, TSCORE, Smoke, Activity at home, Activity at work, Diabetes, CHD, Stroke and COPD. | | | | | | | |

Abbreviations: SD=standard deviation, BMI=body mass index, SYST=systolic blood pressure, TSCORE= the number of SDs the bone density deviates from expected value, CHD=coronary heart disease, COPD=chronic obstructive pulmonary disorder. Activity at work: 1 is sedative, 2 active and 3 very active. Activity at home is active or not.

Significance levels: * p<0.05, ** p<0.01, *** p<0.001

| **Supplementary table 19**. **mtDNA-CN** **risk of M19.** | | | | | | | |
| --- | --- | --- | --- | --- | --- | --- | --- |
| Variable | Person-years,  No. | Cases, No./Persons at risk, No. | Incidence rate, cases/1000 person-years | Incidence rate ratio (95%CI) | HR(95% CI) | | |
|  |  |  |  |  | **Model 1** | **Model 2** | **Model 3** |
| mtDNA-CN *≤ 118.59* | 16109 | 55/1031 | 3.41  (2.62-4.45) | 1 [Reference] | 1 [Reference] | 1 [Reference] | 1 [Reference] |
| mtDNA-CN > *118.59* | 14914 | 58/947 | 3.89  (3.01-5.03) | 1.14  (0.79-1.65) | 1.13  (0.78-1.64) | 1.13  (0.78-1.63) | 1.22  (0.84-1.78) |
| Model 1 crude model. Model 2 adjusted for age. Model 3 additionally adjusted for BMI, SYST, TSCORE, Smoke, Activity at home, Activity at work, Diabetes, CHD, Stroke and COPD. | | | | | | | |

Abbreviations: SD=standard deviation, BMI=body mass index, SYST=systolic blood pressure, TSCORE= the number of SDs the bone density deviates from expected value, CHD=coronary heart disease, COPD=chronic obstructive pulmonary disorder. Activity at work: 1 is sedative, 2 active and 3 very active. Activity at home is active or not.

Significance levels: * p<0.05, ** p<0.01, *** p<0.001

| **Supplementary Table 20. Examples of Power calculations using OpenEPi (**[**https://openepi.com/Menu/OE_Menu.htm**](https://openepi.com/Menu/OE_Menu.htm)**).^32^** | |
| --- | --- |
| **Osteoarthritis** | Using a two-sided 95% confidence interval we may detect a risk ratio of 0.78 for osteoarthritis with 89% statistical power. |
| **Knee osteoarthritis** | Using a two-sided 95% confidence interval we may detect a risk ratio of 0.7 for knee osteoarthritis with 82% statistical power. |
|  |  |
